# The complete mitogenome and plastome of the haptophyte *Pavlova lutheri* NIVA-4/92

**DOI:** 10.1080/23802359.2020.1788436

**Published:** 2020-07-13

**Authors:** Chris J. Hulatt, René H. Wijffels, Kiron Viswanath, Matthew C. Posewitz

**Affiliations:** a Faculty of Biosciences and Aquaculture, Nord University, Mørkvedbukta Research Station, Bodø, Norway; b Department of Chemistry, Colorado School of Mines, Golden, CO, USA; c Bioprocess Engineering, AlgaePARC, Wageningen University and Research, Wageningen, The Netherlands

**Keywords:** Haptophyte, metabolic model, aquaculture, lipid metabolism, DHA

## Abstract

The complete mitochondrial and plastid genomes of the microalga *Pavlova lutheri* strain NIVA-4/92 are reported. The circular-mapping mitogenome is 36,202 bp in length, contains 22 protein-coding genes, 24 *tRNAs*, and has a GC content of 37.5%. Like other haptophytes the mitogenome contains a single large, complex repeat region of approximately 5.4 kbp. The plastome is 95,281 bp in length and has a GC content of 35.6%. It contains 111 protein-coding genes and 27 tRNAs.

The microalga *Pavlova* (Pavlovophyceae) is a rich source of long-chain polyunsaturated fatty acids. Long-used in the aquaculture industry as a live feed, *Pavlova* synthesizes high proportions of docosahexaenoic acid (DHA), eicosapentaenoic acid (EPA), and a set of unique sterols. The Pavlovophyceae comprises a group of four genera and at least 13 characterized species that typically branch some genetic distance from other haptophytes (Liu et al. 2009; Bendif et al. 2011). They have a red-alga derived plastid acquired *via* secondary endosymbiosis, and despite their biogeochemical and industrial significance are under-represented in genomic studies (Baurain et al. 2010). Here we report the complete mitogenome and plastome of *Pavlova* sp. NIVA-4/92, which is available from the Norwegian Culture Collection of Algae (NORCCA) and reportedly originates from Oslofjord, Norway (59°21′N,10°33′E).

High molecular weight DNA was sequenced on a Pacific Biosciences Sequel system by Arizona Genomics Institute (Tucson, Arizona USA). We assembled the whole genome with Canu version 1.7 (Koren et al. 2017), including complete circular-mapping mitochondrial and plastid genome contigs. The sequences were polished to high accuracy with Blasr and Arrow command-line tools from SMRT Link version 5.1 (Pacific Biosciences, Menlo Park, California USA). To ensure there were no remaining indels, 250 bp paired-end Illumina reads were aligned to the genomes with BWA-MEM and the sequences were verified with Pilon (Walker et al. 2014) and FreeBayes (Garrison and Marth 2012). Sequence annotation was assisted by a partial *Pavlova lutheri* mitogenome sequence (HQ908424.1) in addition to GeSeq (Tillich et al. 2017), tRNAscan-SE version 2.0.3 (Chan and Lowe 2019), RNAweasel (http://megasun.bch.umontreal.ca/RNAweasel) and assembled RNA-seq transcripts.

The mitochondrial genome (MN564259.1) is 36,202 bp in length, has a GC content of 37.46%, encodes 22 protein-coding sequences, and 24 tRNAs. It contains a single 5.4 kbp repeat region, a feature found in other haptophyte mitogenomes including *Emiliania huxleyi* (2 kbp repeat region) and *Chrysochromulina* sp. CCMP291 (9.5 kbp repeat region). Analysis with EMBOSS einverted (Rice et al. 2000) indicates that the repetitive region contains a pair of inverted sequences 1846 and 2042 bp in length that share 85.7% identity. Tandem repeats finder (Benson 1999) identified 41 repeat sequences that extend through 5295 bp of the same region. As shown in Figure 1, the genus *Pavlova* forms the outermost branch amongst haptophytes and the mitogenome coding sequences of NIVA-4/92 are identical to those of *Pavlova lutheri* CCMP1325. The plastome of NIVA-4/92 (MT364382.1) is 95,281 bp, has a GC content of 35.60%, contains 111 protein-coding sequences, and 27 tRNAs. Its identity with CCMP1325 confirms that NIVA-4/92 is *Pavlova lutheri*. In connection with the nuclear genome, the mitogenome and plastome sequences will facilitate analysis of organelle bioenergetics, transcription, signaling, construction of compartmentalized genome-scale metabolic models, and potentially aid chloroplast transformation in this industrially significant microalga.

**Figure 1.**
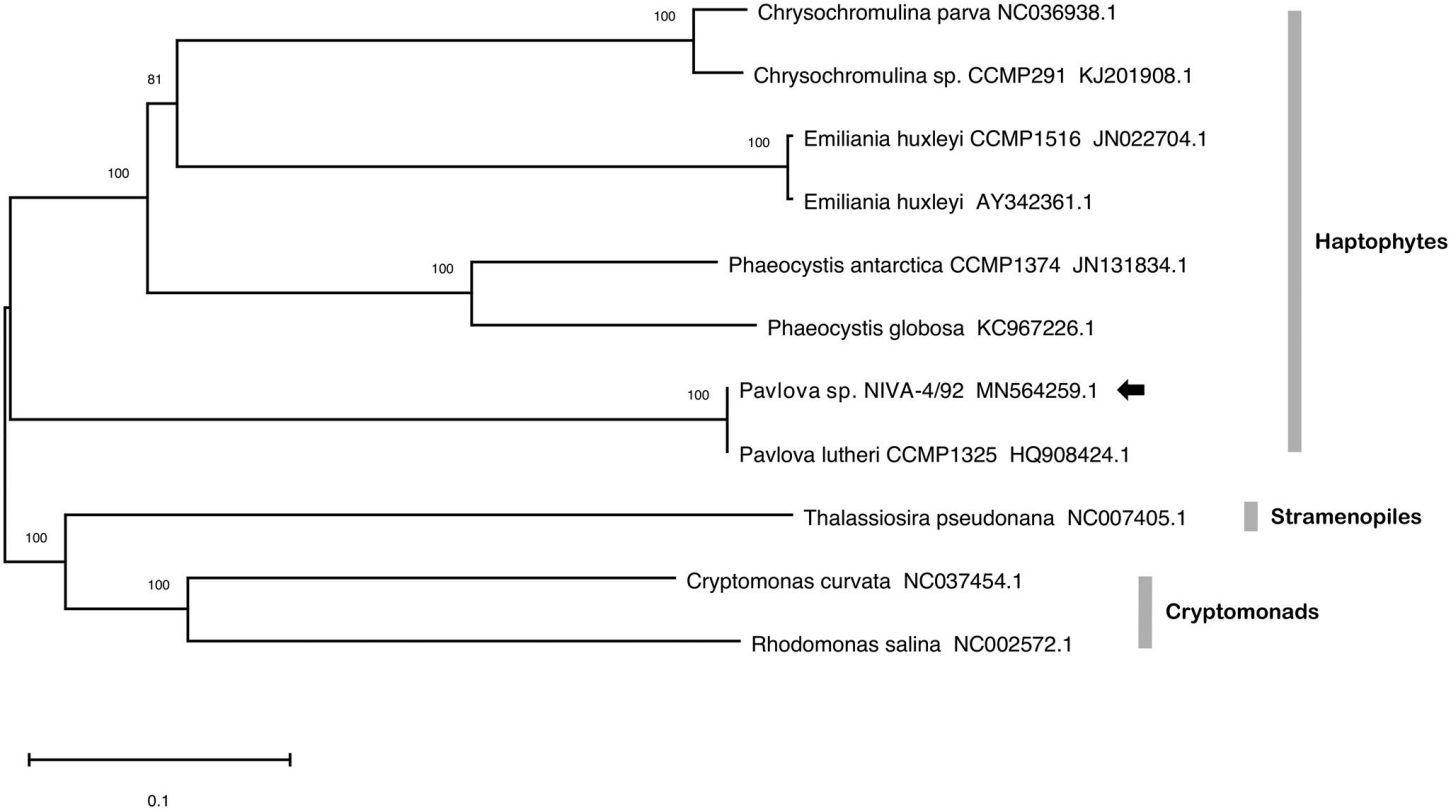
Phylogenetic analysis of 11 mitochondrial genomes with 15 protein-coding genes common to all strains, including eight haptophytes, two cryptomonads, and a diatom. Sequences were aligned with Clustal Omega (Sievers et al. 2011), prepared with GBlocks (Talavera and Castresana 2007), and concatenated to a length of 11,594 nucleotide positions. Tree construction was performed in MEGA-X with neighbor-joining and 1000 bootstrap replications. Units are substitutions per site and support values are indicated.

## Data Availability

The data that support the findings of this study are openly available in GenBank at https://www.ncbi.nlm.nih.gov/genbank/, reference numbers MN564259.1 and MT364382.1.
